# Genetic polymorphisms of *PRKAA1 (AMPKα1)* and postherpetic pain susceptibility: Multicenter, randomized control, and haplotype analysis study

**DOI:** 10.3389/fnmol.2023.1128429

**Published:** 2023-02-01

**Authors:** Yang Mei, Qi Chen, Yu-Ping Li, Yao-hua Chen, Juan Xia, Jie Zeng, Le-hua Yu, Wei Li, Jian Cui

**Affiliations:** ^1^Department of Pain Medicine, Chongqing Hospital of Traditional Chinese Medicine, Chongqing, China; ^2^Department of Pain Medicine, Southwest Hospital, Army Medical University, Chongqing, China; ^3^Department of Anesthesiology, Chongqing University Cancer Hospital, Chongqing, China; ^4^Department of Anesthesiology, The Second Affiliated Hospital of Chongqing Medical University, Chongqing, China; ^5^Department of Rehabilitation Medicine, The Second Affiliated Hospital of Chongqing Medical University, Chongqing, China

**Keywords:** AMPK, *PRKAA1*, polymorphism, postherpetic neuralgia, Chinese population

## Abstract

Adenosine 5′-monophosphate (AMP)-activated protein kinase (AMPK) is a pivotal regulatory protein in energy metabolism. In a pilot study, we found that AMPK-associated energy metabolism imbalance in neurons contributes to the occurrence and maintenance of neuropathic pain (NeP). This study aimed to explore the relationship between genetic polymorphisms of AMPK gene (Rs13361707, rs3792822, and rs10074991) in *PRKAA1* and postherpetic neuralgia (PHN) in Chinese individuals. Hundred and thirty two patients with PHN and 118 control individuals were enrolled in this study. All blood samples were shuffled and blinded to the person performing the haplotype analysis. Rs13361707, rs3792822, and rs10074991 *PRKAA1* genotypes were identified in all participants. Dominant and recessive models were used for evaluating the association between these nucleotide polymorphisms and PHN susceptibility. A haplotype analysis of PHN patients and healthy controls was performed. Clinical characteristics between the two groups were not significantly different (*p* > 0.05) except that the ages in control subjects were younger than the PHN patients (*p* < 0.05). Genotypes and allele frequencies are significantly different between the PHN patients and control subjects for the rs13361707 and rs10074991 polymorphisms (*p* < 0.05), but not for rs3792822 (*p* > 0.05). In addition, the CCG haplotype of rs13361707-rs3792822-rs10074991 correlated negatively with PHN occurrence, but TCA was positively correlated with PHN (*p* < 0.05). Our results indicate that *PRKAA1* gene polymorphisms rs13361707 and rs10074991 were associated with a risk of PHN, and that the CCG haplotype of rs13361707-rs3792822-rs10074991 correlated negatively with PHN occurrence in haplotype analysis. TCA was positively associated with PHN in Chinese individuals.

## 1. Introduction

Postherpetic neuralgia (PHN) is the most common and serious complication after herpes zoster infection. It is a type of intractable neuropathic pain (NeP) that affects mental state and the daily life of patients in severe cases (Mei et al., 2022). The incidence of PHN is approximately 5–30% in patients with herpes zoster (Kawai et al., 2014). However, the exact mechanism of PHN is unclear, and there is a lack of targeted treatment in clinical practice. Accumulating evidence suggests PHN is maintained in part by central sensitization, a phenomenon of synaptic plasticity, and increased neuronal responsiveness in central pain pathways after Herpes zoster infection and painful insults (Hashizume, 2001). A characteristic feature of central sensitization is the activation of glial cells, such as microglia and astrocytes, in the spinal cord and brain, leading to the release of proinflammatory cytokines and chemokines (Meacham et al., 2017). In a pilot animal study, we found the dysfunction of mitochondria play pivotal role in the activation of glial cells and hyperresponsiveness of neurons in spinal dorsal cord (Mu et al., 2022). Dysfunction of mitochondria induced the inefficacy of energy supply and release of proinflammatory cytokines from glial cells, further induced abnormal membrane potential and synaptic plasticity in the vicinity of neurons. Therefore, to further explore its underlying mechanism will help to elucidate the pathogenesis of PHN and to identify effective treatment options.

Adenosine 5-monophosphate (AMP)-activated protein kinase (AMPK) is regulated by the AMP/ATP ratio, and acts on mitochondria as an energy regulator in eukaryotic cells (Lu et al., 2021; Trefts and Shaw, 2021). The AMPK-mTOR signaling pathway plays an important role in regulating cell metabolism, apoptosis, proliferation, and growth. Mutation and inactivation of AMPK can lead to abnormal activation of the mTOR signaling pathway, thus promoting the occurrence and development of neuropathic pain (Krishan et al., 2014; Chen et al., 2022). The expression of phosphorylated AMPK (pho-AMPK) was significantly increased in the spinal dorsal horn and dorsal root ganglia of NeP rats in a chronic constriction injury of sciatic nerve (CCI) model (Cui et al., 2011). Activation of AMPK can improve the energy supply of CCI-damaged nerves, relieve the release of inflammatory factors, and inhibit oxidative stress in the dorsal horn of the spinal cord, thus reducing neuropathic pain (Mu et al., 2022). Three subunits (α, β, and γ) of AMPK were found and the α subunit is a functional unit (Krishan et al., 2014). The α subunit of AMPK contains α1 and α2 subtypes encoded by *PRKAA1* and *PRKAA2* in mammalian cells, respectively (Stapleton et al., 1996). Previous studies have found that gene polymorphisms are closely related to drug metabolism and disease occurrence (Wang et al., 2013; Mei et al., 2015; Guo et al., 2021). However, there are few studies on the correlation between AMPK gene polymorphisms and gastric cancer, rectal cancer, diabetes mellitus and Hepatitis B virus infection (Lee et al., 2014; Yuan et al., 2016; Chen et al., 2018; Jiang et al., 2018; Li et al., 2020). Our previous study identified that the *PRKAA2* gene polymorphism had a significant effect on PHN susceptibility (Mei et al., 2022). The purpose of this study was to explore the association between genetic polymorphisms in *PRKAA1* and PHN in Chinese individuals.

## 2. Materials and methods

### 2.1. Study subjects

The study included 132 patients with PHN and 118 control subjects who were enrolled from the Second Affiliated Hospital of Chongqing Medical University, the Southwest Hospital of Army Medical University, and Chongqing Hospital of Traditional Chinese Medicine. The inclusion criteria were patients with PHN (rash persisting for more than 3 months after clinical healing and pain numerical grade ≥ 4). Healthy controls were randomly selected from the same hospital during the same period for routine health examination. Patients with, kidney, nervous system, liver, or neuromuscular disease were excluded. The study was approved by the Ethics Committee of Chongqing Hospital of Traditional Chinese Medicine (2021-ky-67) and was conducted in accordance with the principles of the Declaration of Helsinki. All subjects provided written informed consent.

### 2.2. Genotyping of PRKAA1 gene polymorphisms

According to the manufacturer’s protocol, we used an SQ blood DNA kit (Omega, Norcross, GA, United States) to isolate the Genomic DNA from peripheral venous blood. Genomic DNA was stored at 4°C (long-term storage at –80°C). All DNA samples were amplified for rs13361707, rs3792822, and rs10074991 by polymerase chain reaction (PCR) using a PCR Amplification Kit (Tsingke Biotechnology, Chongqing, China). The primers for rs13361707 were: forward, 5’-CATTTCAAGCACACCAAGGGC-3′ and reverse, 5’-TGTGTTGCACCATAGATGCTTTT-3′. The primers for rs3792822 were: forward, 5’-GGCTCAAGCCAATTCCGTCA-3′, and reverse: 5’-GGAAGGGTAAGCTGTTCTGC-3′. The primers for rs10074991 were: forward: 5’-CCACACACTCAGTCCCTGAC-3′, and reverse: 5’-AAACCACACGAGCTGGAACA-3′. The PCR products were sequenced by Tsingke Biotechnology Co., Ltd. (Chongqing, China).

### 2.3. Statistics

The sample size was calculated using PASS15 software (Reachsoft, Beijing, China) before the study began based on our previous study, and setting *α* = 0.05, power(1-*β*) = 0.80. Statistical analyses were conducted by SPSS 26.0 (Chicago, IL, United States). The Chi-square test was used to assess the Hardy–Weinberg equilibrium of rs13361707, rs3792822, and rs10074991. The correlation between PHN susceptibility and each single nucleotide polymorphism (SNP), dominant and recessive models, and homozygote and heterozygote comparisons were analyzed by binary logistic regression adjusted for age, BMI, and sex. Linkage disequilibrium and haplotypes were assessed using SHEsis^1^ (Li et al., 2009). Statistical significance was set at *p* < 0.05.

## 3. Results

### 3.1. Subject characteristics

The clinical characteristics including gender, age, body mass index (BMI), mean blood pressure (BP mean), total cholesterol (TCHO), low-density lipoprotein (LDL), high-density lipoprotein (HDL), triglycerides (TG), and fasting blood glucose (FBG) between the two groups was not significantly different (*p* > 0.05), while the healthy subjects were younger than the PHN patients (63.92 ± 13.09 vs. 65.76 ± 9.98, *p* = 0.006; Table 1).

**Table 1:** Basic information of subjects.

	Healthy controls (*n* = 118)	PHN patients (*n* = 132)	*p*-value
Gender (Female/male)	72/46 (156.52%)	76/54 (140.74%)	0.125
Age (years)	63.92 ± 13.09	65.76 ± 9.98	**0.006**
BMI (kg/m2)	23.82 ± 3.13	23.50 ± 2.46	0.525
BP mean (mmHg)	95.67 ± 15.66	93.33 ± 10.30	0.385
TCHO (mmol/L)	4.87 ± 1.21	4.69 ± 0.93	0.353
LDL (mmol/L)	2.89 ± 1.83	2.64 ± 0.78	0.100
HDL (mmol/L)	1.21 ± 0.28	1.22 ± 0.37	0.785
TG (mmol/L)	2.16 ± 2.02	1.68 ± 0.73	0.079
FBG (mmol/L)	7.15 ± 2.40	7.20 ± 2.72	0.917

Basic information of enrolled subjects. Data are given as mean ± SD and were analyzed by independent sample *T* test. BMI, body mass index; BP mean, mean blood pressure; TCHO, total cholesterol; LDL, low-density lipoprotein; HDL, high-density lipoprotein; TG, triglycerides; FBG, fasting blood glucose. Significant results (value of *p* < 0.05) were shown in bold type.

### 3.2. Association study

The genotype and allele frequencies of *PRKAA1* rs13361707, rs3792822, and rs10074991 polymorphisms in patients with PHN and healthy subjects are shown in Table 2. There were significant differences in genotype and allele frequencies between healthy subjects and PHN patients in *PRKAA1* rs13361707 and rs10074991 (*p* < 0.05), but not for rs3792822 (*p* > 0.05). All SNPS were consistent with the Hardy–Weinberg equilibrium (*p* > 0.05).

**Table 2:** Comparisons of genotype and allelic frequencies of *PRKAA1* polymorphisms in PHN patients and healthy subjects.

Genotype	Healthy controls (*n* = 118)	PHN patients (*n* = 132)	*P-*value
rs13361707			
CC	32 (27.1%)	28 (21.2%)	
CT	60 (50.8%)	56 (42.4%)	**0.045**
TT	26 (22.1%)	48 (36.4%)	
Alleles			
C	124 (52.5%)	112 (42.4%)	**0.024**
T	112 (47.5%)	152 (57.6%)	
HWE P	0.831	0.131	
rs3792822			
CC	80 (67.8%)	78 (59.1%)	
CT	34 (28.8%)	46 (34.8%)	0.304
TT	4 (3.4%)	8 (6.1%)	
Alleles			
C	194 (82.2%)	202 (76.5%)	0.118
T	42 (17.8%)	62 (23.5%)	
HWE P	0.869	0.727	
rs10074991			
GG	32 (27.1%)	24 (18.2%)	
GA	60 (50.8%)	58 (43.9%)	**0.018**
AA	26 (22.1%)	50 (37.9%)	
Alleles			
G	124 (52.5%)	106 (40.2%)	**0.006**
A	112 (47.5%)	158 (59.8%)	
HWE P	0.831	0.325	

Data were analyzed by chi-square (*χ*^2^) test. HWE, Hardy–Weinberg equilibrium. Significant results (value of *p* < 0.05) were shown in bold type.

The different analysis models of the *PRKAA1* rs13361707, rs3792822, and rs10074991 polymorphisms on the risk of PHN are shown in Table 3 (all results were adjusted for BMI, age, and sex). In the analysis of rs13361707, the TT genotype showed an increased risk of PHN (CC vs. TT: AOR = 2.108, 95% CI = 1.040–4.272, *P^A^* = 0.039). In the dominant model, using the *PRKAA1* rs13361707 CC + CT genotype as a reference, the TT genotype significantly increased the risk of PHN (CC + CT vs. TT: AOR = 1.939, 95% CI = 1.097–3.426, *P^A^* = 0.023). When the *PRKAA1* rs13361707 CC homozygous genotype was used as a reference, the TT genotype significantly increased the occurrence of PHN (CC vs. TT: AOR = 2.196, 95% CI = 1.066–4.526, *P^A^* = 0.033). When the *PRKAA1* rs13361707 CC homozygous genotype was used as a reference, the CT genotype was not significantly associated with the occurrence of neuropathic pain (CC vs. CT: AOR = 1.136, 95% CI = 0.5955–2.168, *P^A^* = 0.700). There were no significant differences in the recessive model comparison.

**Table 3:** The association between *PRKAA1* genetic polymorphisms and the risk of PHN (adjusted for age, gender, and BMI).

SNPs	Comparisons	AOR (95% CI)	*P^A^* values
rs13361707(C > T)	CC	1.000 (Reference)	
	CT	1.135 (0.601–2.143)	0.695
	TT	2.108 (1.040–4.272)	**0.039**
	CC/CT vs. TT (Dominant model)	1.939 (1.097–3.426)	**0.023**
	CC vs. CT/TT (Recessive model)	1.439 (0.795–2.604)	0.229
	CC vs. TT (Homozygote comparison)	2.196 (1.066–4.526)	**0.033**
	CC vs. CT (Heterozygote comparison)	1.136 (0.595–2.168)	0.700
rs3792822(C > T)	CC	1.000 (Reference)	
	CT	1.454 (0.834–2.535)	0.187
	TT	1.333 (0.545–6.721)	0.311
	CC/CT vs. TT (Dominant model)	1.698 (0.491–5.874)	0.403
	CC vs. CT/TT (Recessive model)	1.506 (0.885–2.562)	0.131
	CC vs. TT (Homozygote comparison)	1.746 (0.476–6.397)	0.400
	CC vs. CT (Heterozygote comparison)	1.415 (0.813–2.415)	0.220
rs10074991(G > A)	GG	1.000 (Reference)	
	GA	1.382 (0.719–2.658)	0.332
	AA	2.601 (1.264–5.352)	**0.009**
	GG/GA vs. AA (Dominant model)	2.086 (1.184–3.672)	**0.011**
	GG vs. AA/GA (Recessive model)	1.763 (0.955–3.258)	0.070
	GG vs. AA (Homozygote comparison)	2.762 (1.315–5.798)	**0.007**
	GG vs. GA (Heterozygote comparison)	1.365 (0.701–2.661)	0.360

Data were analyzed via binary logistic regression. AOR, adjusted odds ratio (adjusted for age, gender and BMI); 95%CI, 95% confidence interval. *P*^A^ adjusted for age, gender and BMI, Significant results (value of *p* < 0.05) were shown in bold type.

In the analysis of rs10074991, the AA genotype increased the risk of PHN (GG vs. AA: AOR = 2.601, 95% CI = 1.264–5.352; *P^A^* = 0.009). In the dominant model, with the GG + GA genotype as the reference, the AA genotype significantly increased the risk of PHN (GG + GA vs. AA: AOR = 2.086, 95% CI = 1.184–3.672, *P^A^* = 0.011). In the recessive model, the GA + AA genotype had no significant difference in the occurrence of PHN as compared with the GG homozygous genotype (GG vs. GA + AA: AOR = 1.763, 95% CI = 0.955–3.258, *P^A^* = 0.070). When the rs10074991 GG homozygous genotype was used as a reference, the AA genotype was found to increase the risk of PHN (GG vs. AA: AOR = 2.762, 95% CI = 1.315–5.798, *P^A^* = 0.007). When the rs10074991 GG homozygous genotype was used as a reference, there was no significant difference between the GA genotype and PHN occurrence (GG vs. GA: AOR = 1.365, 95% CI = 0.701–2.661, *P^A^* = 0.360).

In the analysis of rs3792822, there were no significant differences among genotypes in the recessive model, dominant model, heterozygote comparison, or homozygote comparison (*p* > 0.05).

### 3.3. Haplotype analysis

A linkage disequilibrium (LD) was determined for rs13361707, rs3792822, and rs10074991. The results showed that there was a strong LD among the three SNPs (*D*′ = 0.983, *r*^2^ = 0.921). We used haplotype analysis to study the combination of these three polymorphic loci to assess the risk of PHN. The results showed that CCG and TCA were significantly correlated with the occurrence of PHN in the rs13361707-rs3792822-rs10074991 haploid (Pearson’s *p* = 0.023618). The CCG genotype was associated with a lower risk of PHN [*p* = 0.007, OR 95% CI = 0.609(0.425–0.872)]. TCA genotypes showed an increased risk of PHN [*p* = 0.038, OR 95% CI = 1.491 (1.022–2.176)] (Table 4).

**Table 4:** Haplotype analysis of *PRKAA1* rs13361707, rs3792822, and rs10074991 with the risk of PHN.

	Case(freq)	Control(freq)	Odds ratio [95% CI]	Pearson’s *p*
C C G	101.04 (0.383)	124.00 (0.525)	0.609 [0.425 ~ 0.872]	**0.007**
T C A	96.88 (0.367)	70.00 (0.297)	1.491 [1.022 ~ 2.176]	**0.038**
T T A	52.99 (0.201)	42.00 (0.178)	1.237 [0.788 ~ 1.941]	0.355
C C A	4.08 (0.015)	0.00 (0.000)	–	–
C T A	4.05 (0.015)	0.00 (0.000)	–	–
C T G	2.82 (0.011)	0.00 (0.000)	–	–
T T G	2.14 (0.008)	0.00 (0.000)	–	–

Data were analyzed by online software, SHEsis (Li et al., 2009). Total control = 236.0, total case = 264.0, Global chi^2^ is 7.491477 while df = 2 (frequency < 0.03 in both control and case has been dropped). Pearson’s *p-*value is 0.023618. Significant results (value of *p* < 0.05) were shown in bold type.

## 4. Discussion

We analyzed the *PRKAA1* polymorphic loci rs13361707, rs3792822, and rs10074991. In our study, the variation frequency of rs13361707 in healthy subjects was 72.88%, which was similar to the 71.8% reported by previous research (Li et al., 2020). Otherwise, it was different from the 80.86% reported by Yuan et al. (2016). In our study, the variation frequency of rs3792822 was similar to previous reports (Yuan et al., 2016). The variation frequency of rs10074991 was 72.88% in our study, which differed from the 80.2% reported by Kim et al. (2014). That caused a difference in gene frequency, which might be different from the races that were included in the study. At the same time, gene-region interactions or gene–environment could be another reason for this difference.

In this study, the *PRKAA1* rs13361707 polymorphism was investigated. In the dominant model, the TT genotype had a 1.9 times higher risk of PHN than the CC + CT genotype. Compared with the CC homozygous genotypes, the CT genotypes did not increase the risk of PHN, but the TT genotypes increased the risk of PHN by 2.19 times. In the comparison of the recessive models, no correlation was found between each genotype and PHN. The results of this study suggest that the *PRKAA1* rs13361707 polymorphism is associated with PHN risk. Chen et al. (2018) also found that the *PRKAA1* rs13361707 gene polymorphism was associated with an increased risk of gastric cancer specifically. These results indicated that the *PRKAA1* rs13361707 gene polymorphism may be correlated with some disease occurrence. In the analysis of the *PRKAA1* rs10074991 gene polymorphism, the risk of PHN in the AA genotype was 2.08 times that of GG + GA in the dominant model. Compared with the GG genotype, the AA genotype had a 2.7 times increased risk of PHN. In the recessive model and heterozygous comparisons, there was no significant correlation between the occurrence of PHN and each genotype. The results of this study suggest that the rs10074991 gene polymorphism is associated with disease occurrence, which is similar to previous studies (Kim et al., 2014; Chen et al., 2018). In this study, the AA genotype increased the risk of disease (PHN). However, Chen et al. (2018) found that the GG genotype increased the incidence of the disease, which was similar to Kim et al. (2014)’s findings.

There was no correlation between *PRKAA1* rs3792822 genotypes and PHN occurrence in the dominant model, recessive model, Homozygote comparison, or Heterozygote comparison (*p* > 0.05). However, a recent study showed that rs3792822 polymorphism analyses in both the allele and genotype were associated with the susceptibility of chronic HBV infection (Yuan et al., 2016). Until now, no other studies have reported on the correlation between rs3792822 polymorphisms and disease, so it may be necessary to further explore with future studies.

Haploid analysis was also performed in this study. Theoretically, there should be eight haploid types; however, seven haploid types were detected in this study. This suggests that these alleles are not inherited randomly, but that there is a linkage disequilibrium. *D*′ = 1 and *r*^2^ = 1 are considered complete linkage disequilibria. There is no unified standard for strong linkage imbalance at present, but the generally accepted ones are *D*′ > 0.8 and *r*^2^ > 0.8. In this study, *D*′ = 0.983 and *r*^2^ = 0.921, so we believe that this result is consistent with linkage disequilibrium. Significant differences were observed between these seven haploid types. The TCA haploid significantly increased the risk of PHN, while the CCG haploid significantly reduced the risk of PHN. After completing a literature review, this is the first disease correlation research on rs13361707-rs3792822-rs10074991 haploid type. In previous studies, haploid CTG and TCA of rs13361707-rs1002424-rs3792822 were associated with chronic hepatitis B virus infection (Yuan et al., 2016). Haploid AAGGA of rs1002424-rs2570091-rs10053664-rs13361707-rs3805486 is associated with the development of gestational diabetes mellitus (Guo et al., 2021). This is the first time to reveal the relationship between the difference of haploid types and occurrence of PHN, although it is only a single subtype of a single gene. As more haploid types of other related gene are found, it is believed that haploid analysis will help us to improve the predictive accuracy of PHN occurrence in the future.

In our study, the age of the control group was significantly younger than that of the PHN group (*p* < 0.05). We initially speculated that young people were more likely to be accepted as control subjects, and age was an important independent predictive factor for PHN (Kim et al., 2014). However, careful analysis of the data showed that the age difference between healthy subjects and patients was 1.8 years, and our sample size was large enough to suggest that this result may have been due to casual.

This study had several advantages. This is the first study to investigate the relationship between *PRKAA1* and PHN in Chinese individuals. The *PRKAA1* rs13361707 and rs10074991 polymorphisms were found to be significantly associated with the risk of PHN. Meanwhile, it was found that the haploid TCA and CCG of rs13361707-rs3792822-rs10074991 were closely related to the risk of PHN.

This study had several limitations. First, we studied only the Han Chinese population, and it is not clear that studies in other populations would yield the same results. Second, this study found a close correlation between *PRKAA1* and PHN risk. PHN is a typical neuropathic pain, but it is not clear whether this conclusion can be extended to other types of neuropathic pain (such as painful diabetes). Third, only three polymorphic loci in *PRKAA1* were discussed in this study. Moreover, comprehensive studies are needed to evaluate the correlation between AMPK pathway genes and PHN risk based on SNP polymorphisms and haploid analysis.

Our results indicate that *PRKAA1* gene polymorphisms rs13361707 and rs10074991 were associated with an increased risk of PHN. The CCG haplotype of rs13361707-rs3792822-rs10074991 correlated negatively with PHN occurrence in haplotype analysis, whereas TCA was positively correlated with PHN in Chinese individuals.

## Data availability statement

The raw data supporting the conclusions of this article will be made available by the authors, without undue reservation.

## Ethics statement

The studies involving human participants were reviewed and approved by The Ethics Committee of Chongqing Hospital of Traditional Chinese Medicine (2021-ky-67). The patients/participants provided their written informed consent to participate in this study.

## Author contributions

YM, WL, and JC contributed to conception and design of the study. Y-PL, Y-hC, JX, and JZ organized the database. YM and QC performed the statistical analysis. YM wrote the first draft of the manuscript. YM, QC, L-hY, and JC wrote sections of the manuscript. All authors contributed to manuscript revision, read, and approved the submitted version.

## Funding

This research has been granted by the Chongqing medical scientific research project [Joint project of Chongqing Health Commission and Science and Technology Bureau (2021MSXM006 and 2023MSXM125)], Scientific and Technological Research Program of Chongqing Municipal Education Commission (KJQN202215115), 2021 “Xinglin Scholar” Hospital Special Project of Chengdu University of Traditional Chinese Medicine (YYZX050), Chongqing Scientific Research Institution Performance Incentive and Guidance Special Project (cstc2021jxjl130022), National Natural Science Foundation of China (81870883), and Innovation Project for China Overseas Returnees (cx2017110).

## Conflict of interest

The authors declare that the research was conducted in the absence of any commercial or financial relationships that could be construed as a potential conflict of interest.

## Publisher’s note

All claims expressed in this article are solely those of the authors and do not necessarily represent those of their affiliated organizations, or those of the publisher, the editors and the reviewers. Any product that may be evaluated in this article, or claim that may be made by its manufacturer, is not guaranteed or endorsed by the publisher.

## Footnotes

^1^http://analysis.bio-x.cn
